# Arthritis in Connexion with Uterine Diseases

**Published:** 1849-02

**Authors:** 


*Arthritis in connexion with Uterine Diseases.* By M. Malgaine.—Although it is extremely difficult, or altogether impossible, to explain theoretically the connexion between inflammation of the male urethra and inflammation in remote joints, yet nothing is better proved by observation than the fact of this connexion. So also arthritis, most frequently seated in the knee, is a consequence or concomitant of disease of the uterus. Most authors have looked upon the occurrence of such disease, along with disease of the uterus, as a mere casualty, but M. Malgaigne believes otherwise. At the Hôpital St. Louis he has observed two cases of this description, both of which recovered, after the ordinary treatment for rheumatic arthritis.—*Ibid from Ibid.*

